# HCNet: Multi-Exposure High-Dynamic-Range Reconstruction Network for Coded Aperture Snapshot Spectral Imaging

**DOI:** 10.3390/s26010337

**Published:** 2026-01-05

**Authors:** Hang Shi, Jingxia Chen, Yahui Li, Pengwei Zhang, Jinshou Tian

**Affiliations:** 1 School of Electronics Information and Artificial Intelligence, Shaanxi University of Science and Technology, Xi’an 710021, China; shihang@sust.edu.cn (H.S.); chenjingxia@sust.edu.cn (J.C.); zhangpengwei@sust.edu.cn (P.Z.); 2 The Key Laboratory of Ultra-Fast Photoelectric Diagnostics Technology, Xi’an Institute of Optics and Precision Mechanics of CAS, Xi’an 710119, China; tianjs@opt.ac.cn

**Keywords:** snapshot compressive spectral imaging, multi-exposure fusion, high dynamic range, compressed measurement reconstruction

## Abstract

Coded Aperture Snapshot Spectral Imaging (CASSI) is a rapid hyperspectral imaging technique with broad application prospects. Due to limitations in three-dimensional compressed data acquisition modes and hardware constraints, the compressed measurements output by actual CASSI systems have a finite dynamic range, leading to degraded hyperspectral reconstruction quality. To address this issue, a high-quality hyperspectral reconstruction method based on multi-exposure fusion is proposed. A multi-exposure data acquisition strategy is established to capture low-, medium-, and high-exposure low-dynamic-range (LDR) measurements. A multi-exposure fusion-based high-dynamic-range (HDR) CASSI measurement reconstruction network (HCNet) is designed to reconstruct physically consistent HDR measurement images. Unlike traditional HDR networks for visual enhancement, HCNet employs a multiscale feature fusion architecture and combines local–global convolutional joint attention with residual enhancement mechanisms to efficiently fuse complementary information from multiple exposures. This makes it more suitable for CASSI systems, ensuring high-fidelity reconstruction of hyperspectral data in both spatial and spectral dimensions. A multi-exposure fusion CASSI mathematical model is constructed, and a CASSI experimental system is established. Simulation and real-world experimental results demonstrate that the proposed method significantly improves hyperspectral image reconstruction quality compared to traditional single-exposure strategies, exhibiting high robustness against multi-exposure interval jitters and shot noise in practical systems. Leveraging the higher-dynamic-range target information acquired through multiple exposures, especially in HDR scenes, the method enables reconstruction with enhanced contrast in both bright and dark details and also demonstrates higher spectral correlation, validating the enhancement of CASSI reconstruction and effective measurement capability in HDR scenarios.

## 1. Introduction

Hyperspectral imaging technology can simultaneously capture spatial and spectral information from a scene, exhibiting significant application prospects in fields such as environmental monitoring [1], agricultural detection [2,3], medical diagnosis [4], and cultural heritage diagnosis [5]. The Coded Aperture Snapshot Spectral Imaging (CASSI) [6] technique utilizes a coded mask and a dispersive element to encode and compress three-dimensional (3D) data into a two-dimensional (2D) measurement image. Through compressed sensing algorithms, it reconstructs the 3D data from the 2D compressed measurement, enabling snapshot hyperspectral imaging. Numerous advanced CASSI reconstruction algorithms have been developed, including numerical iterative methods [7,8] and deep learning approaches [9,10,11,12,13,14,15], all of which have demonstrated excellent performance on idealized synthetic datasets. However, practical CASSI systems inevitably encounter factors that significantly degrade reconstruction quality, such as dynamic range limitations. Therefore, it is urgent to develop high-quality hyperspectral imaging methods suitable for real CASSI systems. Regarding dynamic ranges, constrained by the system’s detection scheme and hardware limitations, all spectral channel images are compressed into a single measurement with a finite dynamic range. The recovered hyperspectral images have to segment the dynamic range of the measurement, leading to low-quality reconstruction due to dynamic range shrinking. The more spectral channels involved, the lower the reconstruction quality. Additionally, the system exposure level (overexposure or underexposure) directly determines the data acquisition quality of CASSI. Overexposure causes loss of highlight detail, while underexposure reduces dark-field signal-to-noise ratio (SNR, significantly affected by quantization noise and shot noise), further degrading or invalidating subsequent CASSI hyperspectral image reconstruction.

To address the issue of low-quality reconstruction caused by limited dynamic ranges, inspired by high-dynamic-range (HDR) imaging technology [16,17], this work proposes a high-quality CASSI hyperspectral imaging method based on multi-exposure fusion. Multiple CASSI compressed measurements with different exposure levels are captured to expand the system’s data acquisition dynamic range. Lower-exposure images preserve highlight details, while higher-exposure images improve SNRs in dark regions. The multi-exposure low-dynamic-range (LDR) measurements are then fused to generate an HDR measurement image. Multi-exposure HDR reconstruction networks have matured in RGB imaging (RGB-HDR), widely applied in visual enhancement fields like camera photography and image display, such as Hdr-gan [18], SelfHDR [19], SAFNet [20], Cen-HDR [21], DRHDR [22] and HDRFlow [23]. However, adapting these methods to HDR CASSI compressed measurement prediction presents fundamental differences and challenges. For input data, RGB-HDR reconstruction networks process RGB images containing spatial structures and three color channels, whereas the proposed HDR-CASSI reconstruction network operates on compressed measurement maps—two-dimensional grayscale images resulting from encoding and compressing three-dimensional spatiospectral data. For output targets, RGB-HDR aims to produce visually perception-friendly nonlinear HDR images, whereas HDR-CASSI prioritizes predicting a linear HDR compressed measurement that satisfies physical imaging models. This ensures high fidelity and physical consistency of subsequent hyperspectral reconstruction results. For loss functions, RGB-HDR accommodates visually driven tonal compression, whereas HDR-CASSI must preserve true physical proportions to avoid compromising spatial–spectral consistency.

Therefore, to enhance the imaging quality of real CASSI systems, this work proposes a multi-exposure data acquisition strategy based on CASSI and a multi-exposure fusion-based HDR CASSI measurement reconstruction network (HCNet). Without increasing hardware costs, it can effectively expand the dynamic range of CASSI-compressed measurements through the proposed data acquisition strategy and reconstruction algorithm, providing highly physically consistent HDR compressed measurements for subsequent high-quality hyperspectral image reconstruction. We construct a mathematical model of the multi-exposure CASSI system, comprehensively considering the effects of imaging exposure levels, overexposure clipping effects, and noise on LDR measurements. The composition and working principle of the proposed HCNet is introduced and evaluated by comparing the impact of traditional single exposure versus the proposed multi-exposure strategy on hyperspectral image reconstruction quality. HCNet’s reconstruction robustness under different multi-exposure settings and noise conditions is verified. A real CASSI imaging system is built to validate the high-quality hyperspectral imaging capabilities of the proposed method for scenes with relatively low and high dynamic ranges.

## 2. Methods

**Figure 1:**
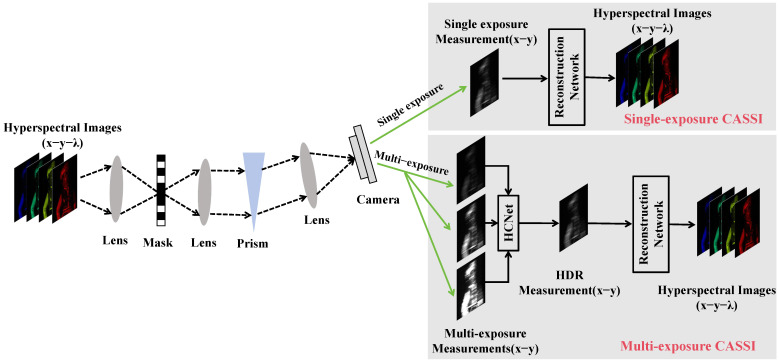
Schematic diagram of single- and multi-exposure CASSI data acquisition strategies.

Figure 1 shows the working principle of the traditional single-exposure and proposed multi-exposure CASSI data acquisition strategies. For a CASSI system, a three-dimensional hyperspectral dataset (x−y−λ) is compressed into a two-dimensional (2D) measurement by spatially encoding, spectrally shifting, and data integrating on a camera. For single-exposure CASSI, a 2D compressed measurement is used to recover hyperspectral images via a hyperspectral reconstruction network. For multi-exposure CASSI, three measurements, captured with different exposure levels, are first fused into an HDR measurement using HCNet, which is then fed into the hyperspectral reconstruction network to provide high-quality hyperspectral images.

To achieve high-quality hyperspectral reconstruction, this section builds upon the single-exposure CASSI model to establish a mathematical description for the proposed multi-exposure strategy, clarifying the objective function and fusion mechanism. Subsequently, the proposed HDR CASSI measurement reconstruction network (HCNet) is described.

### 2.1. Mathematical Model for Single- and Multi-Exposure CASSI

#### 2.1.1. Single-Exposure CASSI

For single-exposure CASSI, the target’s three-dimensional information (x,y,λ) can be represented as a cubic data block $X={\left\{X_{l}\right\}}_{l=1}^{L}$ comprising *L* spectral bands, where $X_{l}\in R^{H\times W}$ denotes the spatial image of the *l*th spectral band.

For a real CASSI system, the camera’s exposure directly affects the number of photons received by the detector, thereby altering the intensity and noise level of the measurement. The exposure can be quantified by the exposure value $EV=\log_{2}\left(G/K\right)$, where *G* is the exposure coefficient and *K* is the reference coefficient. When G=K, EV=0, indicating that the exposure value matches the reference exposure. In this work, G=1 stands for optimal exposure, where the gray values of the output image occupy the full dynamic range without overexposure.

After the spatial modulation with an encoding pattern $C\in R^{H\times W}$, the *l*th spectral image detected by the camera can be expressed as

(1) $$X_{l}^{M}=G\times \left(C⊙X_{l}\right)$$

where ⊙ denotes Hadamard multiplication (element-wise multiplication). Subsequently, the modulated spectral channels undergo dispersion scanning via a dispersive element with distinct imaging positions. The shifted spectral channels overlap on the detector, providing a compressed image $Y\in R^{H^{\prime }\times W^{\prime }}$, which can be expressed as

(2) $$Y=\sum_{l=1}^{L}S_{d}\left(X_{l}^{M}\right)$$

where $H^{\prime }=H$, $W^{\prime }=W+d\left(L-1\right)$, and *d* are the shifted pixels between adjacent channels, and $S_{d}\left(\cdot \right)$ denotes the spatial shift operation.

By considering the inherent shot noise of the detected signal as well as the camera’s limited full well capacity (FWC) and analog-to-digital converter (ADC) bit depth, the detected image can be expressed as

(3) $$Y_{D}=Q\left(SC\left(SN\left(Y\right)\right)\right)$$

where SN(·) denotes the shot noise addition [12], $SC\left(\cdot \right)=\min\left(Y,Y_{max}\right)$ represents the saturation clipping, $Y_{max}$ is the maximum output value of the camera, and Q(·) denotes the quantization via the ADC. Ideally, $Y_{D}$ is the linear combination of all spectral channels, providing an optimal measurement for subsequent hyperspectral reconstruction. However, when the compressed measurement is overexposed, clipping occurs in bright regions, which is nonlinear and irreversible, leading to reconstruction distortion. Additionally, quantization errors obscure subtle contrasts within the compressed measurement, leading to the loss of spatial and spectral details in the recovered images, especially in dark regions.

#### 2.1.2. Multi-Exposure CASSI

To enhance reconstruction quality and robustness, a multi-exposure CASSI measurement strategy is proposed. CASSI measurements with different exposure levels are captured for the same scene. Low exposure preserves bright-region details, while high exposure enhances dark-region contrasts, enabling higher-dynamic-range signal acquisition.

Referring to the common settings for HDR, three exposure levels are adopted, where the medium exposure serves as the reference with EV=0. The exposure coefficients are denoted as $G_{low}$,$G_{mid}$, and $G_{high}$ for low, medium, and high exposures, respectively. According to Equations (2) and (3), the compressed measurements from the limited low-dynamic-range (LDR) camera under the three exposure levels can be expressed as

(4) $$Y_{LDR}^{E}=Q\left(SC\left(SN\left(Y^{E}\right)\right)\right),E=\left\{low,mid,high\right\}$$

where

(5) $$Y^{E}=\sum_{l=1}^{L}S_{d}\;\left(X_{l}^{E}\right),E=\left\{low,mid,high\right\}$$

#### 2.1.3. CASSI Measurement Fusion and Hyperspectral Reconstruction Objectives

Multi-exposure CASSI measurements $Y_{LDR}^{E},E=\left\{low,mid,high\right\}$ are jointly fed into a HDR CASSI measurement reconstruction network for the prediction of an HDR CASSI measurement ${\hat{Y}}_{HDR}\in R^{H^{\prime }\times W^{\prime }}$,

(6) $${\hat{Y}}_{HDR}=f_{\theta }\left(Y_{LDR}^{low},Y_{LDR}^{mid},Y_{LDR}^{high}\right)$$

where $f_{\theta }\left(\cdot \right)$ denotes the HDR CASSI measurement reconstruction network. The predicted ${\hat{Y}}_{HDR}$ is then input into a pretrained hyperspectral reconstruction network to recover the hyperspectral data cube $\hat{X}$.

### 2.2. HDR CASSI Measurement Reconstruction

CASSI-compressed measurements are two-dimensional projections obtained after spatial–spectral encoding and multiplexing. Existing HDR fusion methods are primarily designed for RGB formats and optimized for human visualization, including nonlinear operations such as gamma mapping, while the task of HDR CASSI measurement recovery is to maintain the linear mapping relationship. Ensuring fidelity is the priority, as it directly influences the reconstruction quality and physical interpretability of the hyperspectral images extracted from the HDR CASSI measurement.

**Figure 2:**
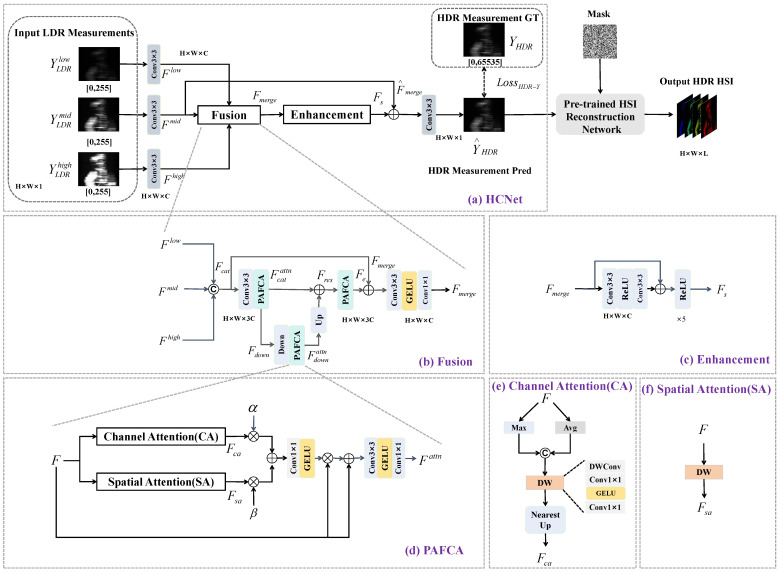
Overview of HDR CASSI-compressed measurement estimation framework for high-quality hyperspectral reconstruction.

To address the issue, an HDR CASSI measurement estimation framework is proposed for high-quality hyperspectral reconstruction. The overview of the framework is shown in Figure 2, composed of an HDR CASSI measurement reconstruction network (HCNet) (Figure 2a) and a pre-trained hyperspectral reconstruction network. Multi-exposure LDR measurements $Y_{LDR}^{E},E=\left\{low,mid,high\right\}$ are first fused by HCNet into an HDR measurement, which is then fed into the pre-trained hyperspectral reconstruction network to produce a high-quality hyperspectral data cube. HCNet prioritizes physical consistency and structural efficiency, comprising a fusion module for preliminary merging of multi-exposure measurements and an enhancement module for feature compression and augmentation. HCNet is constrained using an HDR measurement loss, and the hyperspectral reconstruction network is trained on synthetic HDR measurements without clipping and quantization.

#### 2.2.1. Fusion Module

The Fusion module centers on multi-scale context awareness and channel–spatial attention enhancement, integrating Parallel Adaptive Channel-Spatial Fusion Attention (PAFCA), context enhancement mechanisms, and cross-scale feature interaction. It constructs a feature fusion architecture with context modeling capabilities, as illustrated in the Fusion module block in Figure 2b. Firstly, three LDR measurements undergo 3×3 convolutional feature extraction,

(7) $$F^{E}=Conv_{3\times 3}\left(Y_{LDR}^{E}\right),E=\left\{low,mid,high\right\}$$

The three feature maps $F^{E}$ are concatenated along the channel dimension to form the fused input feature $F_{cat}$. Subsequently, $F_{cat}$ passes through PAFCA to yield $F_{fuse}^{attn}$. PAFCA synergistically employs global channel statistical modeling and local spatial feature perception to adaptively enhance features, emphasizing HDR information in measurements while suppressing redundancy and noise. After attention-enhanced feature fusion, a lightweight single-layer downsampling–upsampling architecture is employed to expand the feature receptive field. Attention-enhanced features undergo downsampling convolution to yield a low-resolution contextual feature $F_{down}$. Similarly, $F_{down}$ passes through PAFCA to obtain $F_{down}^{attn}$, strengthening cross-spatial-scale contextual information. $F_{down}^{attn}$ is then upsampled to its original size via transposed convolution and residually fused with the previous features to yield $F_{res}$. Finally, the fused features undergo progressive enhancement through PAFCA to produce $F_{e}$, ensuring comprehensive and hierarchical feature representation.

To mitigate information loss and maintain feature consistency, the progressively attention-enhanced feature $F_{e}$ is added to the initial concatenated feature $F_{cat}$, forming the cross-stage residual fusion feature,

(8) $$F_{merge}=F_{e}+F_{cat}$$

This residual connection retains the discriminative power of enhanced features while recirculating original information from shallow layers, mitigating potential feature drift or information dilution after multiple processing steps. Finally, the fused feature $F_{merge}$ undergoes further integration of channel information through convolution and activation functions and the output serves as input for subsequent enhancement modules.

#### 2.2.2. Parallel Adaptive Channel-Spatial Fusion Attention (PAFCA)

During multi-exposure feature fusion and context enhancement, efficiently modeling the response distribution within channels and the local–global dynamic characteristics of spatial regions is crucial for suppressing redundancy and enhancing detail preservation and generalization capabilities in HDR measurement prediction. Traditional convolutional attention mechanisms (e.g., BAM [24], CBAM [25]), and existing HDR fusion networks [21,26,27] typically employ compressed vectors from global pooling to model global information. However, local regions in CASSI’s HDR scene maps reflect not only brightness response and exposure intensity distributions but also band-specific information and mask-overlap effects. Simple global statistics struggle to capture these nuances, leading to dilution of critical features. To address this, we designed the Parallel Adaptive Channel-Spatial Fusion Attention Module (PAFCA), as shown in Figure 2d. PAFCA models local–global statistical properties along both the channel (CA) (Figure 2e) and spatial (SA) (Figure 2f) branches, then fuses them within a unified attention adjustment flow. This effectively enhances feature representation and contextual adaptability.

To capture fine-grained statistical distributions, PAFCA first employs localized global pooling. Feature map $F\in R^{C\times H\times W}$ undergoes adaptive average pooling and max pooling with a scale factor *s*

(9) $$F_{avg}=AvgPool_{\frac{H}{s}\times \frac{W}{s}}\left(F\right),F_{max}=MaxPool_{\frac{H}{s}\times \frac{W}{s}}\left(F\right)$$

Unlike traditional global pooling, this $\frac{H}{s}\times \frac{W}{s}$ localized pooling preserves finer-grained statistical features. It reflects global trends within channels while perceiving local dynamic feature distributions. The concatenated channel features undergo local context enhancement through depthwise separable convolutions

(10) $$F_{ca}=DW\left(Concat\left(F_{avg},F_{max}\right)\right)$$

Then, $F_{ca}$ is upsampled back to the original size and fused with the spatial branch features.

The PAFCA spatial branch employs depthwise separable convolutions directly on the spatial dimension to perceive and enhance local structures and spatial variations, yielding $F_{sa}$. Feature results from the spatial and channel branches undergo dynamic adjustment via learnable parameters α and β to produce dual-branch fusion features. The fusion output is activated by GELU and undergoes feature weighting with the original input. It is then processed through a set of convolutional transformations and activations to enhance the expressive power of the features,

(11) $$F_{mod}=\alpha \cdot F_{ca}+\beta \cdot F_{sa}$$

(12) $$F_{out}=F\cdot GELU(F_{mod})+F$$

(13) $$F_{attn}=Conv_{1\times 1}\left(GELU\left(Conv_{3\times 3}\left(F_{out}\right)\right)\right)$$

#### 2.2.3. Enhancement Module

To compress channel information and obtain high-quality HDR measurements, the Enhancement module conducts further feature compression and prediction, as shown in Figure 2c. To reduce subsequent convolutional computations, the fused output features are fed into a 3×3 convolution to compress the multi-channel features back to the original channel number (3C→C). To enhance deep feature representation, supplement local details, and improve network robustness, inspired by the classic ResNet [28] architecture, the compressed features are fed into a ResNet with five stacked residual blocks for successive feature reconstruction:

(14) $$F_{s}=ResNet\left(Conv_{3\times 3}\left(F_{merge}\right)\right)$$

To prevent information drift or degradation during deep feature processing and to incorporate reliable information from the reference measurement, the ResNet output features undergo element-wise residual connection with the medium exposure reference features

(15) $${\hat{F}}_{merge}=F_{s}+F^{mid}$$

To reconstruct high-quality hyperspectral images, a physically consistent HDR measurement is required to avoid distortion. Different from existing HDR reconstruction networks for RGB images, we employ a 3×3 convolution to generate HDR measurements ${\hat{Y}}_{HDR}$ inside of the Sigmoid activation function:

(16) $${\hat{Y}}_{HDR}=Conv_{3\times 3}\left({\hat{F}}_{merge}\right)$$

#### 2.2.4. Loss Function for HDR CASSI Measurement Estimation

Existing HDR networks [20,21,23,27,29,30,31,32] usually employ μ-law mapping for constructing loss functions to reduce the dominance of bright areas in the loss, enhancing the perceptual performance of the overall details. However, this strategy is unsuitable for CASSI systems as the μ-law mapping will introduce strong nonlinear compression in bright regions. Therefore, the $L_{1}$ loss function is employed to provide high-fidelity HDR CASSI measurements for the subsequent hyperspectral reconstruction:

(17) $$L_{HDR-Y}=\frac{1}{N}\sum_{i=1}^{N}|{\hat{Y}}_{HDR}^{i}-Y_{HDR}^{i}|$$

where *i* denotes the *i*th pixel in the image, and *N* is the pixel number of the measurement, $H^{\prime }\times W^{\prime }$.

## 3. Simulation Experiments

### 3.1. Experimental Setup

Two datasets, CAVE [33] and KAIST [34], are utilized for training and evaluation. The CAVE dataset comprises 32 hyperspectral scenes with a spatial resolution of 512×512 pixels and a spectral resolution of 10 nm. It includes 31 spectral channels spanning from 400 nm to 700 nm with an image bit-depth of 16-bit. The KAIST dataset comprises 30 hyperspectral scenes with a spatial resolution of 2704×3376 pixels and a spectral resolution of 10 nm. It includes 31 spectral channels spanning from 400 nm to 700 nm with an image bit depth of 16-bit. Referring to previous CASSI reconstruction methodologies [9,11,12,35], training data is sourced from the CAVE dataset. Training samples are randomly cropped into blocks with 256×256 pixels for data augmentation. Via spectral interpolation, 28 spectral channels spanning from 450 nm to 650 nm are generated. Test data comprises 10 scenes from the KAIST dataset, cropped and interpolated into 256×256×28 hyperspectral data cubes. The default exposure coefficient G=2 (EV=0) is used for medium exposure to include moderate overexposure. For instance, multi-exposure combination EV=[−2,0,+2] corresponds to exposure coefficients of G=[0.5,2,8]. Except for the noise robustness experiment, simulation experiments were conducted under noise-free conditions.

LDR CASSI measurements $Y_{LDR}^{E},E=\left\{low,mid,high\right\}$ are simulated as 8-bit images according to the mathematical model mentioned in Equation (4). The ground truth of the HDR measurement (GT-HDR) is generated with the forward imaging model (Equation (2), G=1) without clipping or quantization, serving as the supervised target for training the proposed HCNet to estimate HDR measurements. Our optimization objective is to minimize the HDR measurement prediction loss $L_{HDR-Y}$ to provide a predicted HDR measurement (Pred-HDR). The classical DAUHST-3stg [9] is adopted as the downstream pre-trained hyperspectral reconstruction network, which is trained using GT-HDR measurements with a $L_{1}$ loss function. The model is constructed using PyTorch 1.12.0 and trained on a single RTX 3090 GPU. Training HCNet for 100 epochs took approximately 3.5 h and achieved convergence. Both HCNet and DAUHST are trained using the Adam optimizer [36] ($\beta_{1}=0.9,\beta_{2}=0.999$) for 300 epochs. The learning rate is initialized to $4\times 10^{-4}$ and scheduled by a cosine annealing strategy.

The evaluation metrics include PSNR, μ-PSNR [37], and SSIM [38]. The results reported in the following simulations are the average values over 10 scenes from the KAIST test dataset.

The peak signal-to-noise ratio (PSNR) is defined as

(18) $$PSNR=10\log_{10}\left(\frac{MAX^{2}}{MSE}\right)$$

where MAX is the maximum pixel value of the image, and MSE is the mean squared error

(19) $$MSE=\frac{1}{N}\sum_{i=1}^{N}{\left(I_{pred}\left(i\right)-I_{gt}\left(i\right)\right)}^{2}$$

where *N* denotes the number of pixels in the image (width × height × number of channels), and $I_{pred}\left(i\right)$ and $I_{gt}\left(i\right)$ represent the reconstructed and ground-truth pixel values at pixel *i*, respectively.

To better preserve details in both dark and bright regions, we adopt the μ-law-modulated PSNR (μ-PSNR), inspired by HDR RGB image quality evaluation methods [18,19,21,32,37,39,40], which is defined as

(20) $$\mu \text{-}PSNR=10\log_{10}\left(\frac{MAX^{2}}{MSE(T_{\mu }\left(I_{pred}\right),T_{\mu }\left(I_{gt}\right))}\right)$$

where

(21) $$T_{\mu }\left(x\right)=\frac{\log(1+\mu \cdot x)}{\log(1+\mu )}$$

where x∈[0,1] is the normalized pixel value and μ>0 is the compression parameter (μ=5000 in this work).

The structural similarity index (SSIM) is defined as

(22) $$SSIM\left(I_{gt},I_{pred}\right)=\frac{\left(2\mu_{I_{gt}}\mu_{I_{pred}}+C_{1}\right)\left(2\sigma_{I_{gt}I_{pred}}+C_{2}\right)}{\left(\mu_{I_{gt}}^{2}+\mu_{I_{pred}}^{2}+C_{1}\right)\left(\sigma_{I_{gt}}^{2}+\sigma_{I_{pred}}^{2}+C_{2}\right)}$$

where $\mu_{I_{gt}}$ and $\mu_{I_{pred}}$ are the mean intensities of $I_{gt}$ and $I_{pred}$, $\sigma_{I_{gt}}^{2}$ and $\sigma_{I_{pred}^{2}}$ are the variances of $I_{gt}$ and $I_{pred}$, $\sigma_{I_{gt}I_{pred}}$ represents the covariance between $I_{gt}$ and $I_{pred}$, and $C_{1}$ and $C_{2}$ are small constants to stabilize the division.

The spectral angle mapper (SAM) is defined as the average angle between the reconstructed spectral vector and the ground-truth spectral vector for each pixel, which evaluates the spectral fidelity of hyperspectral reconstruction:

(23) $$SAM=\frac{1}{N}\sum_{i=1}^{N}\arccos\left(\frac{v_{pred}\left(i\right)\cdot v_{gt}\left(i\right)}{∥v_{pred}\left(i\right)∥\cdot ∥v_{gt}\left(i\right)∥}\right)$$

where $v_{pred}\left(i\right)$ and $v_{gt}\left(i\right)$ represent the spectral vectors (across all *L* spectral bands) at pixel *i* for the reconstructed and ground-truth hyperspectral images, respectively, and ∥·∥ denotes the Euclidean norm. SAM is typically expressed in degrees or radians. The smaller the SAM values, the higher the spectral similarity.

### 3.2. Evaluation of Multi-Exposure Strategies

The performance of the proposed multi-exposure HDR measurement reconstruction framework is evaluated under different exposure combinations and interval settings.

#### 3.2.1. Comparison of Single- and Multi-Exposure Strategies

To our knowledge, no HDR fusion algorithms currently exist for CASSI. Traditional methods and some existing deep learning-based HDR fusion approaches are designed for RGB images. To comprehensively demonstrate HCNet’s superiority and ensure fairness, we compared the hyperspectral reconstruction performances with three single-exposure LDR measurements (low-LDR (EV=−2), mid-LDR (EV=0), high-LDR (EV=+1)), HDR measurements from five existing HDR fusion methods (PFM [41], Cen-HDR [21], DRHDR [22], HDRFlow [23], SAFNet [20]), the Pred-HDR measurement with HCNet (EV=[−2,0,+1]) and the GT-HDR measurement, using the same pretrained hyperspectral reconstruction network.

As shown in Table 1, Pred-HDR significantly outperforms the three single-exposure strategies and five HDR fusion methods in terms of the PSNR, μ-PSNR, SAM and SSIM metrics, approaching GT-HDR’s performance. This demonstrates that multi-exposure fusion effectively mitigates information loss caused by overexposure, underexposure, and LDR. Furthermore, compared to existing HDR fusion networks designed for RGB HDR, our HCNet approach significantly enhances the recovery of spatial and spectral information. The introduction of HCNet increases the computational load of the multi-exposure fusion process by 15.53 GFLOPs and adds 0.63 million(M) parameters.

**Figure 3:**
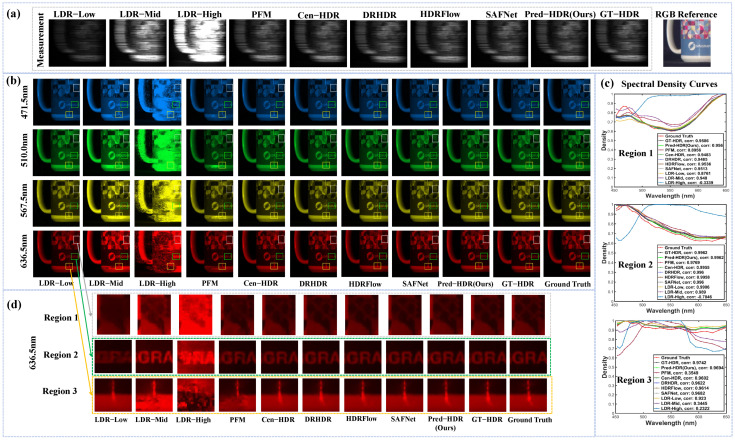
Visualization results of hyperspectral reconstruction using different exposure strategies for the fifth test scene in the KAIST dataset. (**a**) CASSI measurements for single-exposure strategies (low-LDR, mid-LDR, and high-LDR), multi-exposure strategy (PFM, Cen-HDR, DRHDR, HDRFlow, SAFNet, Pred-HDR), and the HDR ground truth (GT-HDR). RGB reference image of the scene. (**b**) Four spectral channel images from the reconstruction results. (**c**) Spectral density curves for the three labeled regions. (**d**) Magnified images of the three labeled regions at 636.5 nm. The white, green, and yellow boxes indicate the selected Regions 1, 2, and 3, respectively.

To visualize the effectiveness of multi-exposure CASSI, reconstructed results for the fifth test scene in the KAIST dataset are shown in Figure 3. Figure 3a shows the CASSI-compressed measurements used for hyperspectral reconstruction and the RGB reference of the scene. Four spectral channels’ images are presented in Figure 3b, where three labeled regions’ spectral density curves are shown in Figure 3c with the spectral consistency metrics (corr). Figure 3d shows the magnified areas of the 636.5 nm channel.

For structures in the three areas (Regions 1–3 labeled with the white, green and yellow boxes, respectively), the results with low LDR exhibit severe detail loss and low contrast. Though more details are recovered with mid-LDR and high LDR, they suffer from reconstruction distortion and artifacts, which are obvious in the spectral density curves. This reveals that the reconstruction performances of the single-exposure measurements are sensitive to exposure levels and the quality will significantly deteriorate when overexposure occurs. Among all compared methods, the results with Pred-HDR are closest to the results with GT-HDR and the ground truth, maintaining high spatial and spectral fidelity. This demonstrates the best recovery of spatial details in Regions 1–3, indicating the proposed method’s superior adaptability and effectiveness in CASSI reconstruction tasks.

To further evaluate the recovery performance of local details, as shown in Table 2, PSNRs and SSIM indexes with different methods for Regions 1–3 (corresponding to Figure 3d) are compared. The results indicate that Pred-HDR with HCNet achieves the best performance across all three regions.

#### 3.2.2. Hyperspectral Reconstruction Performances with Different Exposure Intervals

**Figure 4:**
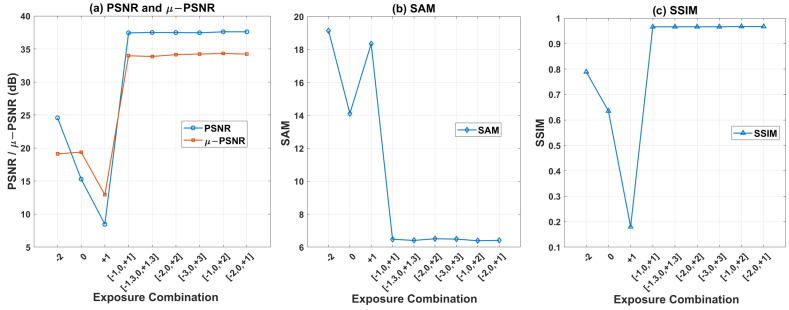
(**a**) PSNR, μ-PSNR, (**b**) SAM, (**c**) SSIM diagrams of hyperspectral reconstruction results for three single-exposure (EV=−2,0,+1) and six multi-exposure (EV=[−1,0,+1], [−1.3,0,+1.3],[−2,0,+2],[−3,0,+3],[−1,0,+2],[−2,0,+1]) CASSI measurements.

The CASSI system has a limited dynamic range. Lower exposures result in more severe underexposure in dark areas, while higher exposures cause overexposure in bright areas. We selected and expanded exposure interval combinations based on experimental settings from Cen-HDR [21] and HDRFlow [23] in RGB HDR research to evaluate our method’s reconstruction performance across different exposure intervals. Figure 4 shows PSNR, μ-PSNR, SAM and SSIM diagrams of hyperspectral reconstruction results for three single-exposure (EV=−2,0,+1) and six multi-exposure (EV=[−1,0,+1],[−1.3,0,+1.3],[−2,0,+2],[−3,0,+3],[−1,0,+2],[−2,0,+1]) measurements.

Multi-exposure strategies outperform single-exposure strategies and are insensitive to exposure interval configurations, demonstrating robust fusion and reconstruction performance for practical experiments with exposure fluctuation.

### 3.3. Loss Functions for HDR CASSI Measurement Prediction

For HDR CASSI measurement prediction, the reconstruction performances are compared with $L_{1}$, $L_{2}$, and $L_{1}$-μ-law loss functions for HCNet, as shown in Table 3. The DAUHST two-stage network [9] serves as the pre-trained HSI reconstruction network. The multi-exposure combination is EV=[−1.3,0,+1.3]. The results demonstrate that measurements recovered using the $L_{1}$ loss function yield superior hyperspectral reconstruction performance, achieving optimal values for PSNR, μ-PSNR, SAM and SSIM. The $L_{2}$ loss function performs slightly worse, while the μ-law-related loss function exhibits further degradation due to dynamic range compression. Therefore, the $L_{1}$ loss function is suitable for HDR CASSI measurement prediction to enable subsequent high-quality hyperspectral reconstruction.

### 3.4. Ablation Experiments

To validate the effectiveness of each key module in HCNet and its adaptation advantages for CASSI, ablation experiments are conducted with stepwise module removal. The pre-trained HSI reconstruction network is the two-stage DAUHST [9]. Here, CA and SA represent the channel branch and spatial branch in PAFCA (Figure 2b), respectively. CA w GP denotes the use of global pooling (GP) in CA, CA w AP denotes the adaptive pooling (AP) in CA, Enhance represents the HCNet’s enhancement module, Sigmoid indicates the Sigmoid activation at HCNet’s output layer, and Act signifies the nonlinear activation after HCNet’s initial feature extraction.

This indicates that introducing either the channel or spatial branch alone (Entries 2, 3, and 4 in Table 4) improves performance, while combining both (5 in Table 4) yields superior results. Adding a residual enhancement module (Enhancement) (6 in Table 4) further improves PSNR and μ-PSNR metrics, demonstrating that residual enhancement effectively optimizes the structure and detail of predicted measurements. Using Sigmoid activation at the output layer (7 in Table 4) or introducing activation operations in the initial stages (8 in Table 4) imposes unnecessary nonlinear compression or disturbance on the feature distribution. This hinders the maintenance of physically consistent measurement predictions and leads to degraded reconstruction performance.

### 3.5. Evaluation of Noise Robustness

To assess the robustness of HCNet under noisy conditions, shot noise is added based on the multi-exposure CASSI imaging mathematical model (Equation (4)). Exposure intervals are set to EV=[−2,0,+1], corresponding to exposure coefficients *G* of [0.5,2,4].

Single-exposure strategies’ measurements are injected with 8-bit shot noise. The noisy LDR measurements are denoted as low/mid/high(noise) LDR. The predicted HDR measurements with the proposed HCNet are denoted as Pred-HDR(noise). GT-HDR measurements include 11-bit shot noise, denoted as GT-HDR(noise).

The experimental results (Table 5) demonstrate that Pred-HDR(noise) exhibits significantly superior reconstruction performance compared to (low/mid/high)(noise) LDR, approaching that of GT-HDR(noise). This indicates that the complementary nature of multi-exposure information partially suppresses noise and enhances hyperspectral image reconstruction quality.

**Figure 5:**
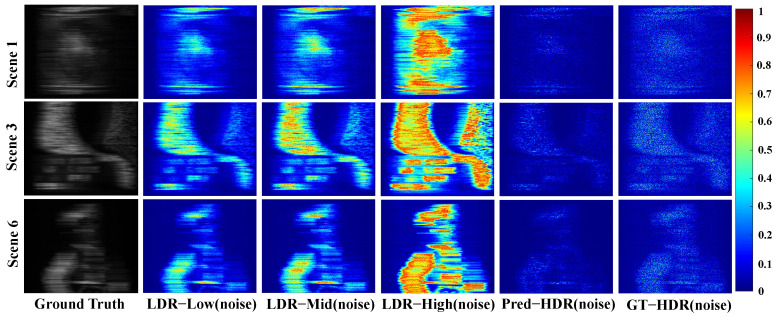
Difference maps ($\left|\hat{Y}-Y\right|$) between noisy measurements ($\hat{Y}$) under various exposure strategies and true noise-free HDR measurements (*Y*) for Scenes 1, 3 and 6 in the KAIST test set.

**Figure 6:**
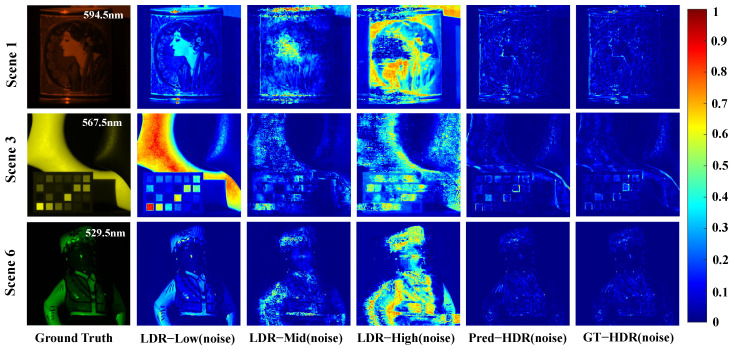
Difference maps $|\hat{X}-X|$ between the hyperspectral reconstruction results ($\hat{X}$) using the CASSI-compressed measurements based on different exposure strategies and the ground truth hyperspectral images (*X*) for the 594.5 nm channel in Scene 1, the 567.5 nm channel in Scene 3, and the 529.5 nm channel in Scene 6.

Figure 5 displays difference maps $\left|\hat{Y}-Y\right|$ between noisy measurements ($\hat{Y}$) and true noise-free HDR measurements (*Y*) for Scenes 1, 3 and 6 in the KAIST test set under various exposure strategies. It is evident that Pred-HDR(noise) exhibits fewer and lower noise compared to the single-exposure LDR noisy measurements. Figure 6 shows the difference maps $|\hat{X}-X|$ between the hyperspectral reconstruction results ($\hat{X}$) using the CASSI compressed measurements based on different exposure strategies and the ground truth hyperspectral images (*X*) for the 594.5 nm channel in Scene 1, the 567.5 nm channel in Scene 3, and the 529.5 nm channel in Scene 6. Results with Pred-HDR(noise) exhibit clearer texture details and more faithful glossiness compared to those from (low/mid/high)(noise) LDR. The reconstructions from low-exposure measurements show significant discrepancies from the ground truth with unrealistic brightness and severe loss of spatial information with medium and high exposure measurements. These results validate the proposed method’s noise robustness and generalization capability for practical CASSI systems with noise.

## 4. Real-World Experiments

**Figure 7:**
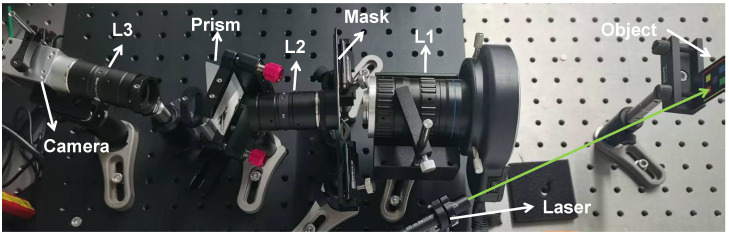
Coded aperture snapshot of spectral imaging system. The green arrow indicates the 516 nm laser beam.

A practical CASSI system was built, as shown in Figure 7, to validate the effectiveness of HCNet for real-world hyperspectral reconstruction. The object is projected on the spatial coding mask with a coupling lens L1. After the spatial modulation, the coded object’s image passes through a 4f system with lenses L2 and L3 (focal lengths f=50 mm), between which a dispersion prism with a top angle of 30° and two filters are placed. The two filters limit the valid detected spectral range from 450 nm to 650 nm. After spectral dispersion and spatial integration, a grayscale camera captures 8-bit CASSI-compressed measurements with a valid area of 760 × 814 pixels. Two scenes are constructed and LDR measurements are captured under low-, medium- and high-exposure conditions with EV=[−2,0,1]. To capture as much of the object’s information as possible, medium exposure serves as a prediction reference, low exposure is not overexposed to preserve the bright details, and high exposure is overexposed to enhance the dark details. Training for the real system uses the CAVE [33] dataset, where training samples are randomly cropped into 380 × 380 pixels blocks for data augmentation.

**Figure 8:**
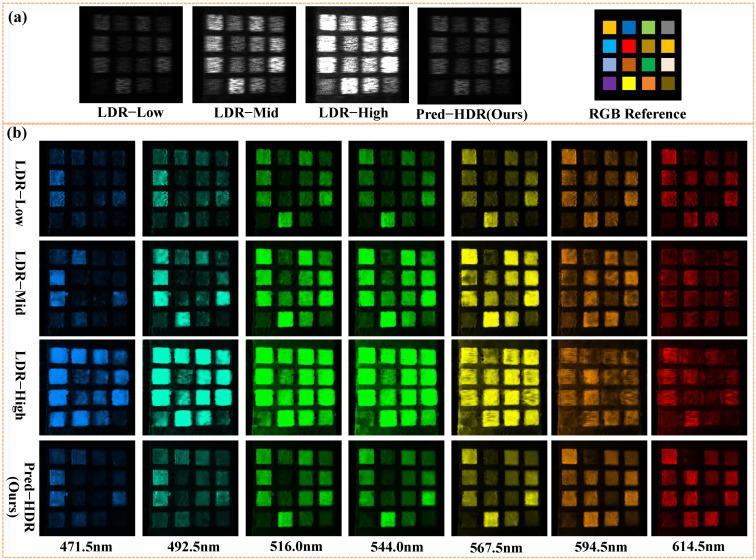
Reconstruction results for Real-World Scene 1. (**a**) Single-exposure measurements, low LDR, mid-LDR, and high LDR. Multi-exposure measurement Pred-HDR with HCNet. RGB reference for Scene 1. (**b**) Recovered hyperspectral images at 7 wavelengths for different exposure strategies. The displayed colors correspond to the RGB visualization of the reconstructed hyperspectral data.

**Figure 9:**
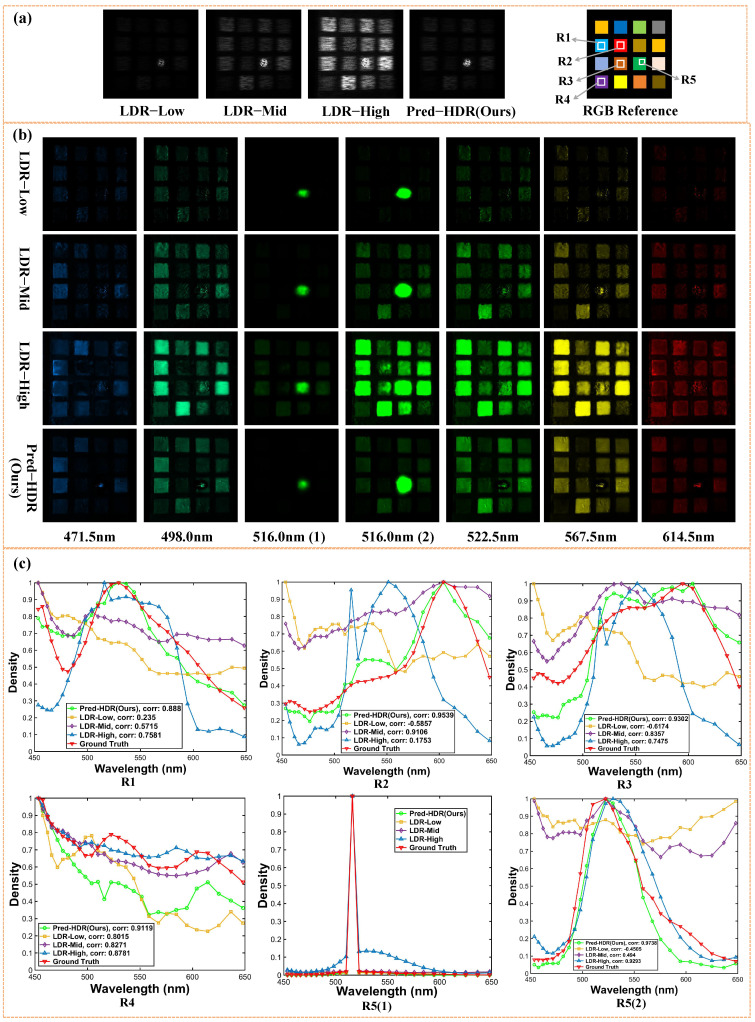
Reconstruction results for Real-World Scene 2. (**a**) Single-exposure measurements, low LDR, mid-LDR, and high LDR. Multi-exposure measurement Pred-HDR with HCNet. RGB reference for Scene 2. (**b**) Recovered hyperspectral images at 6 wavelengths for different exposure strategies. The displayed colors correspond to the RGB visualization of the reconstructed hyperspectral data. The 516.0 nm channel containing the bright laser spot presented with two gray-level ranges, 516.0 nm (1) and 516.0 nm (2). (**c**) Spectral density curves of selected regions R1–R5. R5(1) and R5(2) are the spectral curves with and without the laser.

The reconstruction results for the two scenes are shown in Figure 8 and Figure 9, respectively. Figure 8a shows the three LDR measurements and Pred-HDR measurement with HCNet for Scene 1, whose ground truth is shown as the RGB reference. Figure 8b shows the recovered hyperspectral images at seven wavelengths for different exposure strategies. The reconstruction results with Pred-HDR are visually better than those with the single-exposure strategies, restoring superior details in both bright and dark areas. Although low LDR shows better detail preservation than mid-LDR and high LDR, the spatial contrast is lower than Pred-HDR, suffering from more noise and artifacts. This indicates that it is important to avoid overexposure for traditional single-exposure systems, and the multi-exposure fusion strategy can effectively enhance the reconstruction quality with HDR.

To validate the robustness for higher-dynamic-range scenarios, an HDR scene is constructed by illuminating Scene 1 on the green pattern with a high-power laser (516 nm), as shown in Figure 9. Figure 9a shows the three LDR measurements and Pred-HDR measurement with HCNet for Scene 2, whose ground truth without the laser is shown as the RGB reference. To cover the intensity range from the bight laser spot to the darker patterns, LDR measurements have to be adapted to ensure that the low-LDR measurement is not overexposed and the three LDR measurements follow the optimized exposure interval scheme. Since no similar HDR hyperspectral dataset exists, we simulate the constructed HDR scenes during training by adding a randomly positioned and sized high-intensity Gaussian spot at the 516 nm band in each training sample. Figure 9b shows the recovered hyperspectral images at six wavelengths for different exposure strategies. To clearly demonstrate the HDR imaging performance, the 516.0 nm channel containing the bright laser spot is presented with two gray-level ranges, 516.0 nm (1) and 516.0 nm (2), showing the bright and dark details, respectively. Figure 9c shows the spectral density curves for the selected regions (R1: blue, R2: red, R3: brown, R4: purple, R5: green) labeled in the RGB reference range in Figure 9a.

For laser-illuminated bright regions, mid-LDR and high-LDR loss spatial details exhibit overexposed flat tops, while Pred-HDR restores a Gaussian-shaped spot, which is consistent with the true spatial distribution of the laser (Figure 9b 516.0 nm (1)). For darker background patterns, Pred-HDR performs the best with a higher SNR, less distortion, and fewer artifacts, demonstrating the effectiveness of HDR reconstruction in the spatial domain.

In the spectral domain, for the regions without laser illumination (R1, R2, R3 and R4), spectral density curves with Pred-HDR (green curves in Figure 9c) demonstrate the highest spectral consistency with the ground truth (red) measured using a spectrometer. For the region with laser illumination, Figure 9c R5(1) and R5(2) show the spectral curves with and without the laser, receptively, revealing that Pred-HDR can extract reliable underneath spectral information even with strong interference.

## 5. Conclusions

To address the limitations of reconstructing hyperspectral images in real CASSI systems constrained by the detector’s finite dynamic range, this paper proposes a high-quality CASSI hyperspectral image reconstruction method based on multi-exposure fusion. This method acquires multiple compressed measurements under varying exposure conditions using the CASSI system to capture brighter and darker scene information with a higher dynamic range. In addition, a multi-exposure compressed measurement fusion network (HCNet) is introduced to effectively generate HDR-compressed measurements suitable for CASSI reconstruction tasks, enabling high-quality hyperspectral reconstruction. Unlike traditional HDR reconstruction algorithms focused on visual enhancement, HCNet prioritizes physical consistency as its core design objective, ensuring high fidelity in both spatial and spectral dimensions. Considering real-world system factors such as exposure levels, overexposure clipping, and grayscale quantization, a data acquisition model for the multi-exposure fusion CASSI system is constructed. To validate the proposed method, both simulated and real-world experiments were conducted to compare the hyperspectral image reconstruction quality between multi-exposure and traditional single-exposure strategies. In simulations, HCNet based on multi-exposure fusion effectively generates HDR-compressed measurements, yielding higher-quality hyperspectral reconstructions. Additionally, it demonstrates high robustness against small exposure interval shifts and shot noise, making it suitable for real experimental systems. In real-world experiments, the CASSI experimental system was built to shoot two scenes with relatively low and high dynamic ranges. The proposed method consistently demonstrates optimal reconstruction quality, including higher-contrast spatial details and more coherent spectral information, validating its effective measurement capability in real-world HDR scenarios.

Compared to single-exposure CASSI reconstruction pipelines, the proposed HCNet exhibits increased computational complexity due to the incorporation of multi-exposure fusion. The experimental results demonstrate that embedding HCNet or existing HDR algorithms exhibits increased GFLOPs and parameter counts. Although this entails additional computational costs during training, experiments reveal that the testing time with the pre-trained HDR fusion network increases by only approximately 0.1 s compared to single-exposure strategies. This enables direct deployment in real-time imaging applications, delivering superior HDR-compressed measurements and significantly enhancing hyperspectral reconstruction quality for HDR scenes. Future work will focus on exploring lightweight network architectures to reduce computational complexity during training while maintaining high reconstruction performance.

Furthermore, the current framework assumes that multi-exposure measurements are obtained from static or quasi-static scenes with neglectable spatial movement during the data acquisition process. For dynamic scenes with significant spatial position variations, this remains a challenge and can be explored in the future with regard to network improvements and system optimizations.

## Figures and Tables

**Table 1:** Comparison of the hyperspectral reconstruction performances (PSNR, μ-PSNR, SSIM, SAM, GFLOPs, Params) with three single-exposure LDR measurements (low-LDR (EV=−2), mid-LDR (EV=0), high-LDR (EV=+1)), HDR measurements from five HDR fusion methods (PFM, Cen-HDR, DRHDR, HDRFlow, SAFNet), the Pred-HDR measurement with HCNet (EV=[−2,0,+1]), and the GT-HDR measurement. Boldface indicates the best results in each column except for GT-HDR.

Method	PSNR (dB)	μ-PSNR (dB)	SSIM	SAM	GFLOPs	Params (M)
LDR-Low	24.583	19.124	0.789	19.145	**20.105**	**1.379**
LDR-Mid	15.291	19.393	0.635	14.113	**20.105**	**1.379**
LDR-High	8.466	12.902	0.181	18.352	**20.105**	**1.379**
PFM	33.901	27.741	0.930	9.893	21.917	1.425
Cen-HDR	36.753	29.021	0.953	8.754	24.958	1.582
DRHDR	37.472	33.397	0.964	6.915	86.711	2.567
HDRFlow	37.383	32.936	0.964	7.052	31.552	1.644
SAFNet	37.487	33.413	0.966	6.987	71.291	2.492
Pred-HDR	**37.585**	**34.224**	**0.967**	**6.411**	35.632	2.013
GT-HDR	37.734	34.513	0.969	6.185	20.105	1.379

**Table 2:** Comparison of local PSNRs and SSIM indexes (upper and lower values in cells) with different methods at 636.5 nm for Regions 1–3 (Figure 3d) in the fifth scene of the KAIST dataset. Boldface denotes the best results in each column except for GT-HDR.

Method	Region 1	Region 2	Region 3
LDR-Low	18.99 (0.652)	21.19 (0.668)	16.21 (0.781)
LDR-Mid	8.86 (0.552)	11.89 (0.588)	11.23 (0.361)
LDR-High	2.57 (0.173)	5.05 (0.153)	7.64 (0.006)
PFM	30.68 (0.858)	30.91 (0.940)	20.08 (0.718)
Cen-HDR	35.31 (0.936)	33.96 (0.969)	31.26 (0.949)
DRHDR	36.89 (0.955)	35.49 (0.977)	30.92 (0.950)
HDRFlow	36.64 (0.952)	35.01 (0.975)	30.96 (0.947)
SAFNet	37.27 (0.957)	35.56 (0.977)	31.01 (0.951)
Pred-HDR	**37.28** **(0.957)**	**35.57** **(0.977)**	**32.11** **(0.958)**
GT-HDR	37.58 (0.961)	36.42 (0.981)	31.94 (0.958)

**Table 3:** Hyperspectral reconstruction performances with different loss functions for HCNet. Boldface denotes the best results in each column.

Loss Function	PSNR	μ-PSNR	SAM	SSIM
$L_{1}$	**36.68**	**33.19**	**8.85**	**0.961**
$L_{2}$	36.37	31.55	8.98	0.958
$L_{1}$-μ-law	34.84	32.38	9.51	0.947

**Table 4:** Hyperspectral reconstruction performances for ablation experiments. Boldface denotes the best results in each column.

		PSNR	SSIM	μ-PSNR	SAM	GFLOPs	Params
1	Baseline	36.15	0.954	31.57	9.21	**15.15**	**0.942**
2	1 + CA w GP	36.35	0.957	32.36	9.02	23.01	1.423
3	1 + CA w AP	36.51	0.959	32.89	8.99	23.09	1.425
4	1 + SA	36.61	0.960	32.99	8.91	26.38	1.228
5	3 + 4	36.63	0.961	33.07	8.86	28.10	1.527
6	5 + Enhance	**36.68**	**0.961**	**33.19**	**8.81**	30.68	1.573
7	6 + Sigmoid	36.22	0.956	32.17	9.33	30.97	1.585
8	6 + Act	36.18	0.955	31.85	9.55	31.08	1.601
9	7 + 8	36.27	0.955	32.35	9.54	31.38	1.616

**Table 5:** Comparison of hyperspectral reconstruction performances for noise robustness evaluation. Boldface denotes the best results in each column except for GT-HDR.

Method	PSNR	μ-PSNR	SAM	SSIM
LDR-Low (noise)	21.51	16.08	16.03	0.578
LDR-Mid (noise)	12.14	14.58	18.01	0.483
LDR-High (noise)	8.75	13.51	18.35	0.241
Pred-HDR (noise)	**33.44**	**30.98**	**9.36**	**0.926**
GT-HDR (noise)	34.15	31.31	9.08	0.933

## Data Availability

The raw data supporting the conclusions of this article will be made available by the authors on request.
